# Diphtheria Analogous to Erysipellatous Inflammation

**Published:** 1878-12

**Authors:** W. O’Daniel

**Affiliations:** Bullards, Ga.


### DIPHTHERIA ANALOGOUS TO ERYSIPELLATOUS INFLAMMATION.

By W. O'DANIEL, M.D., of Bullards, Ga.

I have for many years in my own experience noticed the identical anology of diphtheria with erysipelatous inflammations. Pseudomembraneous croup (mostly confined to children) and putrid sore throat are in some respects synonyms of diphtheria. The word diphtheria, coming from a Greek word, signifying membrane. Every practitioner of medicine has no doubt been horrified at the ravages of this most fatal disease in its malignant form. Diphtheria was known, perhaps, to the early writers, but not by this name, until so called by Bretonneau, perhaps half a century ago.

The croupous form has had the greatest fatality among children. While I desire to record my experience in a few cases, I am not *entirely* satisfied that such analogy is general, but of sufficient frequency to have had my attention attracted to certain pothological phenomena which have caused me to regard the very striking similarity or analogy in the two diseases. I have carefully noted a few cases of erysipelatous inflammation supervening diphtheria when a subsidence of the diphtheretic exudation and symptoms *immediately* commenced.

I remember one case in a little girl, six years old, which I considered hopelessly ill with diphtheria, when a sore came upon the foot which was considered insignificant by myself and the parents of the child—so much so that treatment was considered unnecessary. This turned out to be a malignant crysipelatous inflammation which progressed rapidly. I then thought I had a dreadful complication to contend with, which would, of course, have had a tendency to lessen the chances for recovery, but to my utter surprise and almost inexpressible gratification, in a few hours a noticeable subsidence of the inflammation of the throat and fauces commenced, and improvement continued until a speedy and complete recovery ensued.

I remember two other cases in adults, in which the same results were manifest. One was a white lady about 40 years old, who had an obstinate case of diphtheria, when an unmistakable erysipelas commenced in the arm and rapidly progressed with very unfavorable symptoms. In this case the throat symptoms very rapidly subsided, the erysipelas crontrolled and the lady had a good recovery.

The other was a servant girl of my friend and professional brother, Dr. S. L. Richardson, who fell under my professional care during the doctor's absence from home. She, too, had malignant diphtheria. I treated her two or three days when the doctor's return made further attention from me unnecessary, and I consequently lost sight of the case.

In consultation with the doctor some time afterwards, he casually remarked to me that "The girl you treated took erysipelas in the face which extended rapidly to the scalp," when the diphtheria subsided and the girl had a speedy recovery.

I have mentioned these cases out of many; I do not mean to convey the idea that all cases of diphtheria are thus associated with erysipelas, but that it does frequently occur, and that the same toxemia *may* produce these conditions.

If it is true that a metastatic influence thus palliates or controls in any way diphtheria in the slightest degree, could we not reasonably expect similar results from counter-irritation made at will? Now, in regard to the treatment I would say, that after purgatives (if necessary) and rest, I rely mostly on the internal use of the mur. tr. iron and quinine as the *chief* remedies indicated. I frequently use lotions and washes of carbolic acid and the dilute mur. tr. iron. I prescribe the iron, largely diluted with water, and given almost to saturation, and the quinine until the specific effect is had. Of course, always being governed by circumstances, and treating symptoms as they arise. This plan of treatment has given me better satisfaction than any I have yet adopted.

---

